# GroupRank: Rank Candidate Genes in PPI Network by Differentially Expressed Gene Groups

**DOI:** 10.1371/journal.pone.0110406

**Published:** 2014-10-16

**Authors:** Qing Wang, Siyi Zhang, Shichao Pang, Menghuan Zhang, Bo Wang, Qi Liu, Jing Li

**Affiliations:** 1 Department of Bioinformatics & Biostatistics, School of Life Science and Biotechnology, Shanghai Jiao Tong University, Shanghai, China; 2 Department of Biomedical Informatics, Vanderbilt University School of Medicine, Nashville, Tennessee, United States of America; 3 Center for Quantitative Sciences, Vanderbilt University School of Medicine, Nashville, Tennessee, United States of America; 4 Shanghai Center for Bioinformation Technology, Shanghai, China; Memorial Sloan Kettering Cancer Center, United States of America

## Abstract

Many cell activities are organized as a network, and genes are clustered into co-expressed groups if they have the same or closely related biological function or they are co-regulated. In this study, based on an assumption that a strong candidate disease gene is more likely close to gene groups in which all members coordinately differentially express than individual genes with differential expression, we developed a novel disease gene prioritization method GroupRank by integrating gene co-expression and differential expression information generated from microarray data as well as PPI network. A candidate gene is ranked high using GroupRank if it is differentially expressed in disease and control or is close to differentially co-expressed groups in PPI network. We tested our method on data sets of lung, kidney, leukemia and breast cancer. The results revealed GroupRank could efficiently prioritize disease genes with significantly improved AUC value in comparison to the previous method with no consideration of co-exprssed gene groups in PPI network. Moreover, the functional analyses of the major contributing gene group in gene prioritization of kidney cancer verified that our algorithm GroupRank not only ranks disease genes efficiently but also could help us identify and understand possible mechanisms in important physiological and pathological processes of disease.

## Background

It remains a big challenge to detect associations between diseases and genes although many disease candidate genes haven been reported through genetic studies such as linkage analysis [1] and association studies [2]. Prioritizing genes according to their likelihood of being disease genes using computational methods can help biologists find the most promising candidate genes for further downstream verification. Many tools have been developed, most of which use a guilt-by-association concept that ranks highest candidate genes similar to known disease genes.

Among them, Endeavour is a well-developed tool that ranks the candidates against the profile of the training set of genes known to be involved in a biological process or a disease of interest, combining 20 data sources such as functional annotations, expression data, regulatory information, literature, pathways, interactions, sequence, and disease probabilities [3], [4]. In a variety of data sources, fast accumulating protein-protein interaction (PPI) data is a valuable resource for gene prioritization because the genes tend to be highly connected in the protein-protein interaction network when they are related to a specific biological function or similar disease phenotype [5]. Some tools have been developed to perform gene prioritization using this network and have performed well, including CGI [6], GeneWanderer [7], and DIR [8]. For example, comprising the interactions from HPRD [9], BIND [10], BioGrid [11], IntAct [12] and DIP [13], GeneWanderer ranks candidate genes using a global network distance measure and random walk analysis for the definition of similarities to known disease genes in protein-protein interaction networks.

But the gene prioritization methods that measure the similarities to known disease genes by guilt-by-association or network distance cannot be applied accurately for a rare or even unknown disease gene. Recently, some efforts have been made to combine PPI network and global gene expression to conduct gene prioritization, the assumption of which is that nodes neighboring to differentially expressed genes are disease gene candidates [14], [15]. The advantage of this kind of methods is that no prior knowledge about the biological process or disease genes is needed as a training set. However, we found that there is a risk of high false positive rates and low robustness when candidate genes are close to only a single gene with dramatic change in expression.

Genes usually show co-expression if they have the same or a closely related biological function or are co-regulated by the same transcript factor. In order to prioritize disease genes more precisely and robustly, we proposed a new algorithm called GroupRank to rank disease genes by integrating PPI network and gene groups clustered by coordinately differential expression. Our assumption is that, as well as differentially expressing in cases and controls, a strong disease gene candidate is more likely close to gene groups in which all members coordinately differentially express than to individual ones. To verify this assumption and evaluate the performance of our method, we applied GroupRank into the gene expression datasets of four cancer types including lung, kidney, leukemia and breast cancer.

## Materials and Methods

### Gene expression data collection

Four microarray gene expression datasets of humans in case-control design were downloaded from the NCBI Gene Expression Omnibus (GEO) [16] for lung cancer (GSE12428), kidney cancer (GSE6344), leukemia (GSE10631), and breast cancer (GSE29270). All these datasets were curated and reported in the GEO Datasets (GDS). More details about these datasets were summarized in Table S1.

### Cancer gene list

We collected disease genes of lung, kidney, leukemia, and breast cancer respectively from OMIM [17] and Cancer Gene Census [18] (see Table S2). The OMIM database provides the connections between genes and lots of diseases. Cancer Gene Census is an ongoing effort to catalogue those genes for which mutations have been causally implicated in cancer.

### PPI network

We used HINT as a protein-protein interaction network that is a database of high-quality protein-protein interactions in different organisms (http://hint.yulab.org/) [19]. These PPI links have been compiled from different sources and then filtered both systematically and manually to remove erroneous and low-quality interactions. There are 27493 binary and 7629 co-complex interactions in HINT for *H.sapiens.*

### Differential expression analysis

The statistical analysis of gene differential expression was computed by Student t-test and Bonferroni correction was applied. Only the genes having a corrected p-value less than 0.05 remained in the following gene grouping.

### Gene grouping

In GroupRank, we first clustered the differentially expressed genes into the co-expressed groups. We defined the distance between two genes by 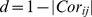. Here 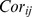 represents the Pearson correlation coefficient of the expression of gene iand gene j. Then, hierarchical clustering was applied to partition the differentially expressed genes into groups. The sizes and the number of groups are changed by adjusting the threshed of gene distance d within a group from 0 to 1.

### Performance measurement

We measured the performance of ranking algorithms using the method described by Zhao *et al* (2011) [15]. Briefly, for a known disease gene in a candidate gene set of size N, if the predicted ranking position is r, then the rank ratio r/N may reflect how well this gene is ranked as a disease gene by our algorithm. Lower rank ratio represents better predictive performance. Optimized parameters could be determined through minimizing the average rank ratio of all known disease genes. In addition, we applied the receiver operating characteristics (ROC) analysis [18] [20] to evaluate the overall performance.

### Algorithm of GroupRank

First, we defined the similarity matrix of genes by adopting *discrete diffusion kernel* from the Diffusion Rank algorithm reported by Yang et al. [21].

As described in Pinta [14], the transition probability matrix W of a random walk on a given graph G is defined as 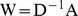. A is the adjacency matrix and D is the diagonal matrix of G. Consider 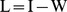, and then we obtain the similarity matrix of genes
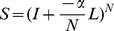
(1)where parameter 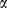 is the diffusion rate, and N is the number of iterations. In this paper, we set 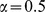 and 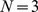 as the previous studies found that few iterations is sufficient to reach a considerably good performance [14], [22].

Then, from the genes differentially expressed in cancer and normal control, we classify them into co-expressed gene groups by hierarchical clustering. When ranking a candidate gene using a gene group 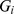 in the PPI network, we define the rank score of a candidate gene obtained from group 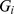 as
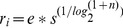
(2)where s is the similarity score between the candidate gene and group 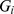, which is measured with the geometric mean of the values in the similarity matrix S between the candidate gene and each member in group 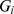. Parameter e represents the differential expression level of the gene group, which is computed by the geometric mean of log2 ratio (cancer/control) of each gene within the group G. n is the group size.

In the analysis of the active gene subnetwork of disease, highly connected nodes are often penalized and the size of the subnetwork is controlled [23]. To avoid bias and control possible false positives in the gene ranking that result from either the super group containing large numbers of gene members or the extremely high degree of the candidate gene itself as a hub in the PPI network, we adopted the method of Gaire et al [23] and added adjustable penalization parameters 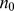 and 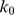 into the following modified formula (3):
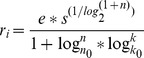
(3)k is the degree of the candidate gene in network HINT. The smaller 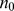 and 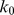 are, the more stringent penalization is carried to the hub genes and the co-expressed group with super-size. Since the mean degree of network HINT is 6.7, we set 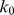 = 15, and 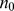 = 20 as default values in this paper.

Finally, the integrated ranking score of a candidate gene contributed by all gene groups is calculated as
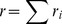
(4)


## Results and Discussion

### Performance evaluation

**Figure 1:**
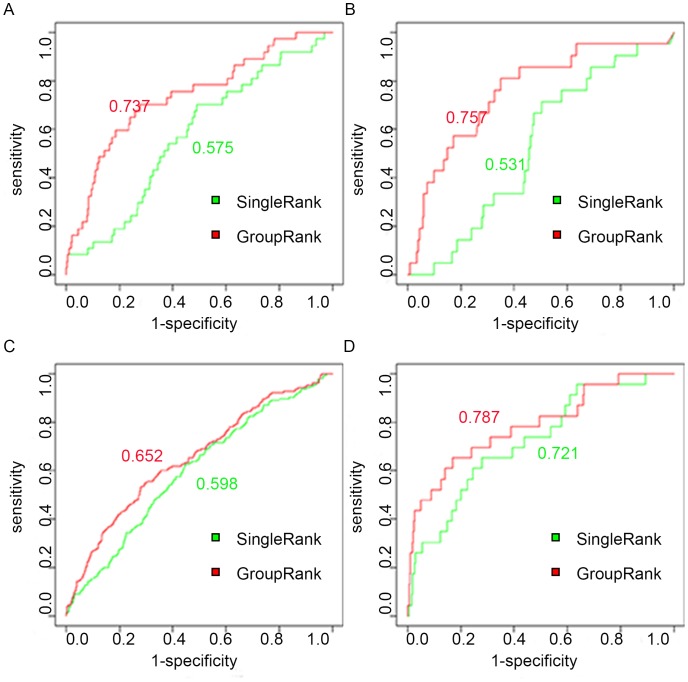
Mean rank ratio of GroupRank using different distance thresholds. The gene groups in GroupRank are partitioned based on a distance threshold with a gradient from 0.1 to 0.9. From A to D, the cancer types are lung cancer, kidney cancer, leukemia and breast cancer.

We tested GroupRank in four cancer related microarray datasets (lung, kidney, leukemia, and breast cancer) individually. Mean rank ratio (MRR) of known disease genes predicted by our algorithm was used to evaluate its overall performance. By adjusting the threshold of distance d from 0 to 1 with the gradient of 0.01 in defining a gene group, the best MRR was obtained when an optimized threshold was chosen (Table 1). In the results, the best thresholds of distance for different cancer types fell into 0.2–0.6 (Figure 1). A possible explanation is that using a more rigorous threshold, there are not enough effective groups that can be formed, while all genes are possibly classified into very few super groups with poor correlations if a more relaxed threshold is applied.

**Table 1:** MRR of GroupRank in four cancer types.

Cancer type	Lung cancer	Kidney cancer	Leukemia	Breast cancer
MRR	0.257	0.227	0.298	0.192
Optimized distance	0.42	0.60	0.52	0.24

**Figure 2:**
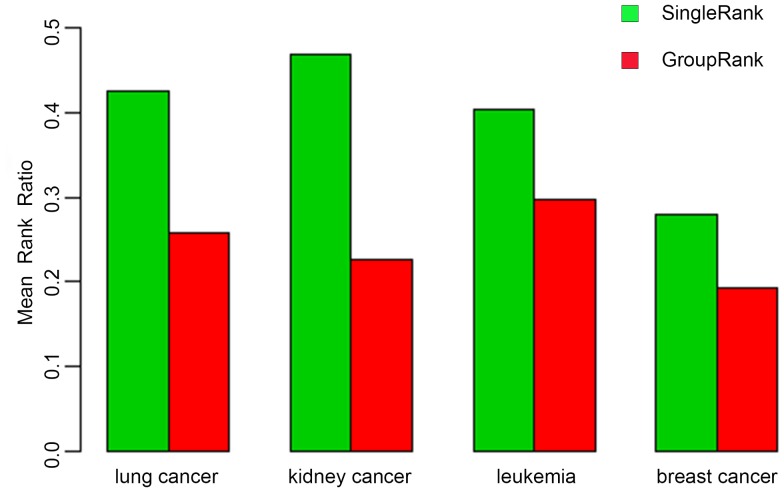
MRR comparisons of GroupRank and SingleRank. The colored bar chart shows the mean rank ratio (MRR) in disease gene ranking using GroupRank (red) and SingleRank (green). It indicates that GroupRank performs better with a lower MRR (p-value<0.001).

We compared the performance of GroupRank with the previous similar method that ranks candidate genes based on individual expressed genes in the PPI network [14]. To distinguish from GroupRank, we called this method SingleRank. As the results show in Figure2, the MRR of known disease genes predicted using GroupRank was lower than SingleRank in each testing dataset when a fixed distance threshold of 0.5 was used. We ran a paired Wilcoxon test and revealed that the improvement of MRR brought by GroupRank algorithm was significant (p-value<0.001).

**Figure 3:**
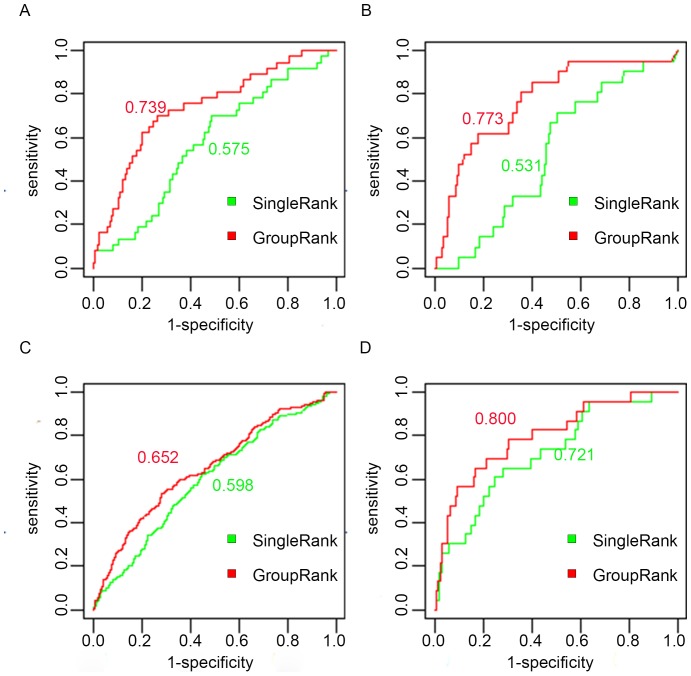
ROC curves of GroupRank and SingleRank. Performance validation using ROC curves. The AUC values of GroupRank and SingleRank achieved in each cancer type are labeled. From A to D, the cancer types are lung cancer, kidney cancer, leukemia and breast cancer.

Additionally we plotted ROC curves to compare GroupRank and SingleRank. Figure 3 shows that GroupRank achieved AUC values from 0.65 to 0.80 in four cancers, which were higher than the values from SingleRank. The results suggest that GroupRank using co-expressed gene groups is a more efficient approach than the method simply using the individual genes in disease gene ranking. It implies that gene prioritization by gene groups could reduce noise and achieve better accuracy.

### Grouping Efficiency by co-expression

**Figure 4:**
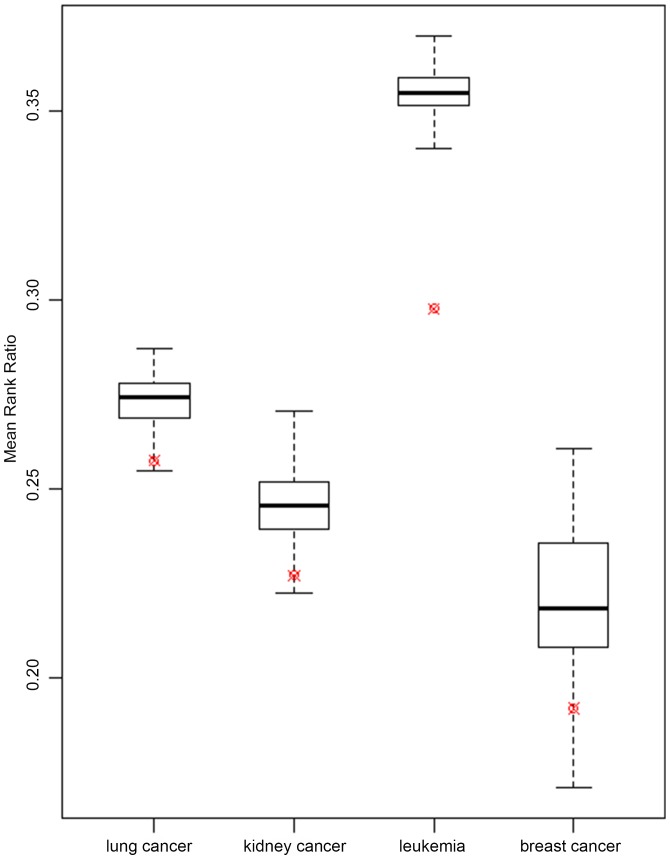
MRR Comparisons of GroupRank using co-expressed and random groups. The red sign represents MRR of GroupRank using co-expressed gene groups in four cancers. Boxplots show the distributions of MRRs using random groups of the same size. The random sampling was repeated 1000 times in each cancer type.

In the GroupRank algorithm, we assumed that the differentially co-expressed gene groups are surrounding a good disease gene and thus are effective to rank disease gene candidates. In order to validate this assumption, we compared the ranking performance using co-expressed and random gene groups. The random groups having the same size were generated by randomly sampling from the PPI network. We repeated the sampling 1000 times. The results indicate that, in all four cancers we studied, the mean rank ratios using co-expressed groups are significantly better than using random gene groups (p-value<0.05) (see Figure 4). It suggested that the downstream genes of a strong disease gene tend to be co-expressed into a number of groups.

### Major contributing groups in gene ranking

**Figure 5:**
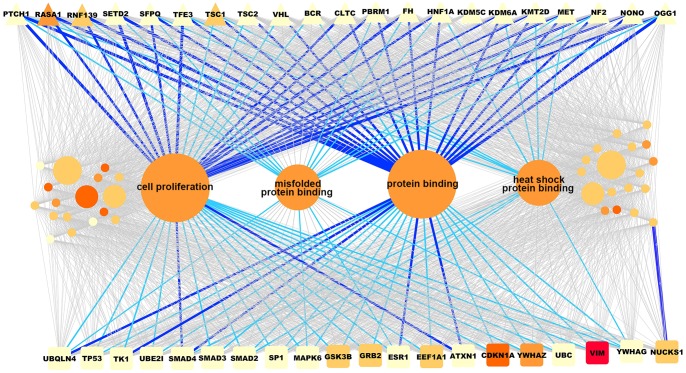
Schematic graph of gene ranking of kidney cancer using GroupRank. The graph illustrates gene ranking of kidney cancer using the algorithm GroupRank. The triangle nodes at the top represent known kidney cancer genes and the square nodes at the bottom represent the top 20 ranked genes of kidney cancer using GroupRank. The circle nodes in middle represent the co-expressed gene groups used to rank disease gene candidates. A known or putative cancer gene is connected with a gene group if it contributes more than 5% of the summed ranking score of this cancer gene. The width of the edge linked to a disease gene is proportional to the scoring contribution obtained from the corresponding gene group. The edges explaining more than 20% of the ranking score of the cancer gene candidate are highlighted in dark blue. The edge is colored in light blue if the scoring contribution of the gene group is from 15% to 20%. The darker node color indicates higher fold change at expression level in cancer and normal control. The size of the circle node representing gene group was proportional to its accumulated contribution in ranking scores of all known kidney cancer genes. The enriched functional annotation is labeled on each of the four major contributing gene groups.

In the GroupRank algorithm, the co-expressed gene groups comprising the most significantly changed gene members in cancers and normal controls must play major roles in cancer. Looking at it from another angle, further study on those major contributing groups can help us to explore and understand why a candidate gene is listed in the top rank and which pathway or biological process is influenced by this disease gene candidate in the disease condition. In this paper, kidney cancer was taken as an example, and we investigated the gene groups, especially the major contributing groups in the ranking of the top 20 gene candidates and 21 known kidney cancer genes. As illustrated in Figure 5, based on the accumulated contributions in ranking scores of known tumor genes using GroupRank, four gene groups emerged by explaining 64.7% of the ranking scores of all 21 known kidney cancer genes. We found that the top 20 ranked genes also had strong connections with those four groups. That indicates that these four gene groups are closely related with kidney cancer. We did GO enrichment analysis of these groups using WebGestalt [24] and found that these gene groups, which were differentially expressed in kidney cancer, are involved in cell proliferation, protein binding, misfolded protein binding, and heat shock protein binding respectively (p-value<0.05, bonferroni multiple testing adjustment). It was reported by Short *et al*. (1993) that enhanced cell proliferation occurs at several stages of renal tumorigenesis [25]. Heat shock proteins (Hsps) are overexpressed in a wide range of human cancers and are implicated in tumor cell proliferation, differentiation, invasion, metastasis, death, and recognition by the immune system [26]. Misfolded proteins were also reported in the study of cancer, and targeted degradation of misfolded proteins has become one of the promising new therapeutic approaches in the treatment of cancer [27].

## Conclusion

In this study, by combining PPI network and gene differential expression and co-expression data, we proposed a new algorithm GroupRank, in which disease candidate genes were ranked by the surrounding differentially co-expressed gene groups in PPI network. The results demonstrated that GroupRank could improve the accuracy of disease gene prioritization significantly. Furthermore, the further functional analysis of the major contributing groups in ranking may not only help us predict disease gene candidates but also improve the biological interpretation of data.

## Supporting Information

Table S1 The list of microarray gene expression datasets.
(DOC)

Table S2 Cancer gene list.
(DOC)
