# Supplementary material for: GroupRank: Rank Candidate Genes in PPI Network by Differentially Expressed Gene Groups
Source: PLoS One. 2014 Oct 16;9(10):e110406. doi: 10.1371/journal.pone.0110406 (PMC4199715; doi:10.1371/journal.pone.0110406)
Supplement: Table S1 — The list of microarray gene expression datasets. (DOC) [file pone.0110406.s001.doc]

**Table S1 The list of microarray gene expression datasets**

| Cancer type | GEO accession | Platform | Reference |
| --- | --- | --- | --- |
| Lung cancer | GSE12428 | GPL1708 | PMID: [19334046](http://www.ncbi.nlm.nih.gov/pubmed/19334046) |
| Kidney cancer | GSE6344 | GPL96, GPL97 | PMID: [20502531](http://www.ncbi.nlm.nih.gov/pubmed/20502531) |
| Leukemia | GSE10631 | GPL96 | PMID: [18477771](http://www.ncbi.nlm.nih.gov/pubmed/18477771) |
| Breast cancer | GSE29270 | [GPL4133](http://www.ncbi.nlm.nih.gov/geo/query/acc.cgi?acc=GPL4133) | NA |
