# Supplementary material for: GroupRank: Rank Candidate Genes in PPI Network by Differentially Expressed Gene Groups
Source: PLoS One. 2014 Oct 16;9(10):e110406. doi: 10.1371/journal.pone.0110406 (PMC4199715; doi:10.1371/journal.pone.0110406)
Supplement: Table S2 — Cancer gene list. (DOC) [file pone.0110406.s002.doc]

**Table S2 Cancer gene list**

| Cancer type | Cancer genes (Entrez gene ID) |
| --- | --- |
| Lung cancer | 5071,673,3791,1326,6385,5979,11186,6597,2064,5519,10568,3799,5925,5002,4610,5290,10342,7080,9940,207,7170,7430,3845,4221,8314,121227,27436,6098,1956,972,6657,238,80304,2263,8030,6794,4780,3659 |
| Kidney cancer | 8643,4841,8085,7249,29005,4771,7403,7428,29072,2271,5727,55193,8289,5921,7942,6927,7248,7030,1213,6608,5546,8242,4233,11236,4968,84925,6421 |
| Leukemia | 23092,6964,8805,286530,4763,2177,4869,6777,1050,54880,1045,4683,1387,27,6427,8233,2120,51517,1785,1021,5159,4515,4893,7704,10801,10962,171023,868,92521,27004,345930,865,2000,6455,8028,4629,6887,4926,23624,80312,3131,675,5187,3205,23305,861,64901,6955,4300,2006,3229,3209,4026,171017,64919,3927,4004,8522,4291,3207,9321,3195,11168,7955,2122,10019,6184,9623,2624,863,4609,3227,8833,596,894,5551,124540,613,63976,2060,6957,7913,3092,4005,3845,2175,2188,84295,27125,5108,79728,4066,3239,3718,1662,2033,1788,2176,83990,23048,10978,5087,54904,6491,2322,51119,6886,595,5396,25925,604,55294,602,64783,3815,3899,4297,10006,2623,5914,862,23157,867,2189,2178,8021,53335,7514,3716,7307,10272,8115,2521,6146,64109,10320,5413,30012,405,150465,6929,57591,26511,10215,641,27086,25,3110,5781,3492,3717,5079,10499,7150,3237,4330,2213,4851,2078,3932,607,5927,5910,23365,4928,8301,6946,8148,694,64324,8030,3575,6418,23522,29844,5371,472,57082,9135,603,7994 |
| Breast cancer | 2033,207,9821,83990,8142,8493,7157,2625,2064,3845,4916,55193,672,8314,595,5925,675,999,79728,5002,2120,5290,7251,11200,2099,6416 |
